# US Veterans Show Improvements in Subjective but Not Objective Sleep following Treatment for Posttraumatic Stress Disorder: Secondary Analyses from a Randomised Controlled Trial

**DOI:** 10.1155/2023/7001667

**Published:** 2023-08-14

**Authors:** Danielle C. Mathersul, R. Jay Schulz-Heik, Timothy J. Avery, Santiago Allende, Jamie M. Zeitzer, Peter J. Bayley

**Affiliations:** ^1^School of Psychology, Murdoch University, Murdoch, WA 6150, Australia; ^2^Centre for Molecular Medicine and Innovative Therapeutics, Health Futures Institute, Murdoch University, Murdoch, WA 6150, Australia; ^3^War Related Illness and Injury Study Center (WRIISC), Veterans Affairs Palo Alto Health Care System, Palo Alto, CA 94304, USA; ^4^Department of Psychiatry and Behavioral Sciences, Stanford University School of Medicine, Stanford, CA 94305, USA; ^5^Mental Illness Research, Education and Clinical Center (MIRECC), Veterans Affairs Palo Alto Health Care System, Palo Alto, CA 94304, USA

## Abstract

**Background:**

Sleep disturbances are a prominent feature of posttraumatic stress disorder (PTSD), and poorer sleep quality is associated with higher PTSD severity. This highlights the importance of monitoring sleep outcomes alongside PTSD symptoms in treatments targeting PTSD. Yet few studies monitor both sleep and PTSD outcomes, unless sleep is the primary treatment target. Furthermore, inconsistencies remain about the effects of first-line, evidence-based PTSD treatments on sleep.

**Methods:**

Here, we explored changes in sleep in secondary analyses from a randomised controlled trial that originally assessed the noninferiority of a breathing-based yoga practice (Sudarshan kriya yoga; SKY) to a first-line PTSD treatment (cognitive processing therapy (CPT)) for clinically significant PTSD symptoms among US veterans (intent-to-treat *N* = 85; per protocol *N* = 59). Sleep was assessed via subjective (self-reported sleep diary), PTSD symptom severity items (self-reported and clinician-administered insomnia/nightmare sleep items), and objective (wrist actigraphy) measures.

**Results:**

Following treatment, subjective sleep diary measures of quality, latency, and wake duration showed small effect size (*d* = .24 − .39) improvements, with no significant differences between treatment groups. Significant improvements were also observed in PTSD sleep symptoms, though CPT (*d* = .34) more reliably reduced nightmares while SKY (*d* = .44-.45) more reliably reduced insomnia. In contrast, there were no significant treatment-related effects for any of the actigraphy-measured sleep indices.

**Conclusions:**

To our knowledge, this is the first study to investigate sleep as an outcome of CPT or SKY for PTSD, across a combination of subjective diary, PTSD symptom severity, and objective actigraphic measures. Findings lend support to a growing body of evidence that trauma-focused psychotherapy for PTSD improves sleep and suggest that yoga-based interventions may also be beneficial for sleep among individuals with emotional or mental health disorders like PTSD. This trial is registered with NCT02366403.

## 1. Introduction

Sleep disturbances are a prominent feature of posttraumatic stress disorder (PTSD) and appear in two of the five diagnostic symptom clusters (category B, nightmares; category E, difficulty sleeping (insomnia); [1]). Not surprisingly, most individuals with PTSD have sleep disturbance [2–4]. Sleep disturbances are also the most commonly reported symptom among individuals who have been exposed to a traumatic event yet do not meet full PTSD criteria [2, 5, 6]. Indeed, insomnia and PTSD symptoms are bidirectionally related over time [7], and poorer sleep quality is associated with higher subsequent PTSD severity [6, 8]. Meta-analyses of objective biomarkers of sleep (e.g., polysomnography, actigraphy) are consistent with the high frequency of self-reported sleep disturbances in PTSD [9, 10]. These findings have led to the notion that sleep disturbances are a core feature of PTSD, beyond mere secondary symptoms [11].

Clearly, it is important to monitor sleep outcomes alongside PTSD symptoms in treatments targeting PTSD. Yet few studies do, unless sleep is the primary treatment target. A recent systematic review and meta-analysis across pharmacological, psychological, behavioural, and complementary and integrative health interventions for PTSD found only 89 randomised controlled trials (RCTs) out of 2139 full-text publications reported a sleep outcome [12]. Overall, interventions significantly improved both sleep and PTSD outcomes, with those specifically targeting sleep the most successful at improving sleep outcomes. However, the extant literature remains inconclusive regarding the effect of *first-line PTSD treatments* on sleep. On closer inspection of these 89 studies, only four [13–16] delivered a first-line, evidence-based treatment for PTSD (e.g., cognitive processing therapy (CPT), prolonged exposure therapy (PE), trauma-focused cognitive behavioural therapy (TF-CBT), or eye-movement desensitisation and reprocessing (EMDR)) [17–22]. Of these four studies, two demonstrated significant improvements in self-reported sleep [13, 16]—though neither achieved full remission of sleep disturbances—while the other two studies found significant residual sleep disturbances despite improvements in PTSD severity [14, 15]. Furthermore, the two studies reporting improvements in sleep [13, 16] used a self-report measure specifically designed to assess sleep quality (e.g., a sleep diary or standardised questionnaire), while the other two studies [14, 15] relied solely on the insomnia and nightmare items from PTSD symptom severity measures. Together, these inconsistencies and limitations across only a handful of studies with very different sample demographics—females only [13], chronic PTSD [15], or actively serving military/defence personnel [14]—highlight the need for further research.

An overreliance on self-report measures of sleep exposes findings to bias [23], whether these are sleep-specific or subsumed within PTSD assessment. The most relevant biases to treatment studies are social desirability and demand characteristics, where participants may adjust their responses for specific reasons (e.g., to be less personally disclosing or to appear “better” or “worse” at the end of treatment). For sleep research specifically, memory recall is another likely potential bias. Using objective measures alongside subjective self-report measures is an important way of increasing ecological validity. Yet, to our knowledge, no study to date has examined *both* subjective *and* objective sleep within the context of a treatment study for PTSD. Here, we address multiple gaps in the literature by examining *both* subjective *and* objective sleep in US veterans undergoing first-line PTSD treatment (CPT) for clinically significant PTSD symptoms. Sleep was assessed via subjective reporting (a standardised, valid, and reliable self-reported sleep diary), PTSD symptom severity items (insomnia/nightmare sleep items on self-reported and clinician-administered PTSD measures), and objective measures (validated wrist actigraphy). This combination addresses the extant literature inconsistencies and limitations, such as differences in the type of sleep measure (some used standardised sleep diaries/questionnaires, others used PTSD insomnia/nightmare sleep items, and we used both), failure to deliver a first-line, evidence-based treatment for PTSD (we used CPT), and an overreliance on subjective, and self-reported sleep (we used objective actigraphy).

To determine whether any sleep changes were specific to trauma-focused treatment or might occur following other interventions, we explored these sleep changes as secondary analyses from an RCT that originally assessed the noninferiority of a breathing-based yoga practice (Sudarshan kriya yoga (SKY)) to CPT. As the primary outcomes study found SKY was clinically noninferior to CPT for symptoms of PTSD, depression, and negative affect at the end of treatment, we hypothesised that all sleep measures (self-reported, clinician-administered, and actigraphy) would improve with both CPT and SKY for PTSD. We also examined differences in sleep between treatment groups, though based on the limited extant literature, we did not pose directional hypotheses.

## 2. Methods

### 2.1. Participants

US veterans with clinically significant levels of PTSD symptoms (≥38 on the PTSD Checklist for DSM-5; PCL-5; [24]) were recruited from the San Francisco Bay Area via flyers and advertisements. In total, 85 veterans with PTSD (59 treatment completers) were randomised to treatment [25, 26]. As per the recommended rigorous approach to testing noninferiority [27], here, we report both intent-to-treat (ITT; *N* = 85) and per protocol (PP; ≥75% treatment sessions; *N* = 59) secondary exploratory analyses on the sleep data. Approximately 14% and 9% of sleep actigraphy data were lost at baseline and end-of-treatment, respectively, due to poor data quality. Demographics by treatment group sample are presented in Table 1 (additional demographic data are available in the primary outcomes study; [25]). The presence of sleep apnoea and restless legs were assessed via two well-validated self-report measures with high sensitivity and specificity: the Multivariate Apnea Prediction Index (MAPI; [28]) and Restless Legs Syndrome Diagnostic Index (RLS-DI; [29]), respectively. The recommended cut-off for the MAPI is .50, suggesting our sample likely had sleep apnoea. Negative scores on the RLS-DI suggest our sample did not have restless legs syndrome. Groups did not differ on basic demographics (all *p* > .05). The CONSORT 2010 checklist of information for RCT is presented as a Supplementary File (available here).

### 2.2. Procedure

The study was approved by the Stanford University Institutional Review Board. The full RCT protocol is described elsewhere [25, 26]. Briefly, US veterans who had clinically significant PTSD symptoms were randomised to receive 6 weeks of either CPT (individual, twice-weekly) or SKY (initial 5-day group workshop, then 5 weeks of twice-weekly groups), per recommendations and protocols for each intervention [25, 26]. Multiple clinician-administered, self-report, and physiological measures were administered at multiple timepoints. Here, we report sleep measures taken at two timepoints: baseline and end-of-treatment. All overnight sleep assessments were taken on weekday nights.

### 2.3. Measures

#### 2.3.1. PTSD Sleep Symptoms

The afternoon before the overnight objective sleep assessment, participants completed several self-report questionnaires and clinician-administered measures, including the Posttraumatic Stress Disorder Checklist–Civilian Version (PCL-C) [32] and Clinician-Administered PTSD Scale for DSM-5 (CAPS-5) [33]. The PCL-C was the primary outcome measure for our RCT and is a well-validated measure that assesses DSM-IV PTSD symptom severity in the last month on a 5-point Likert scale from 1 “not at all” to 5 “extremely”, such that higher scores indicate greater severity and scores of 3 “moderately” or more on individual items indicate clinically significant severity. We chose the PCL-C over the PCL-5 as our primary outcome measure for the original noninferiority RCT because, at the time of commencement, there was no established threshold for clinically significant (or noninferiority) improvement for the PCL-5 [25, 26]. Instead, the PCL-5 was used as a screening measure to ensure clinically significant levels of PTSD symptoms for inclusion.

Per previous studies [14, 16], we obtained the PTSD sleep symptom values from the PCL-C for self-reported *nightmares* (item 2: “Repeated, disturbing dreams of a stressful experience from the past?”) and *insomnia* (item 13: “Trouble falling or staying asleep?”). The CAPS-5 is the gold standard, semistructured, clinical diagnostic interview for DSM-5-defined PTSD. Clinicians rate each symptom item on a scale from 0 “absent” to 4 “extreme/incapacitating,” such that higher scores indicate greater severity and scores of 2 “moderate/threshold” or more on individual items indicate clinically significant severity. Per previous studies [13, 15, 16], we obtained the PTSD sleep symptom values from the CAPS-5 for *nightmares* (criterion B2: “In the past month, have you had any unpleasant dreams about (EVENT)?”) and *insomnia* (criterion E6: “In the past month, have you had any problems falling or staying asleep?”).

#### 2.3.2. Objective Sleep (Actigraphy)

Following completion of the PTSD symptom measures, participants were fitted with a Motionlogger wristwatch (Ambulatory Monitoring, Ardsley, NY) that records triaxial accelerometer (actigraphy) data and asked to wear the device until they returned for their follow-up assessment the next morning. We collected a single night's sleep at each timepoint (baseline, end-of-treatment) and used validated algorithms [34] embedded in manufacturer-provided software (ActionW, Ambulatory Monitoring, Ardsley, NY) to define established quantitative sleep variables. Specifically, we extracted sleep *fragmentation* (objective index of quality; measure of sleep interruptions due to physical movement), *latency* (time to sleep onset), duration (total sleep time in mins), *wake duration* (total duration in mins of time awake after sleep onset), and *efficiency*(percentage of total sleep time relative to total time in bed) to correspond with the self-reported sleep diary indices.

#### 2.3.3. Subjective Sleep (Diary)

The morning following the overnight objective sleep assessment, participants completed the Core Consensus Diary, a standardised, valid, and reliable self-report measure of nightly subjective sleep [35]. From question responses, we obtained values for self-reported sleep quality (“How would you rate the quality of your sleep?” (1 = very poor, 2 = poor, 3 = fair, 4 = good, and 5 = very good)), *latency* (“How long did it take you to fall asleep?”), *duration* (calculated as time between “What time did you try to go to sleep?” and “What time was your final awakening?” minus *latency* minus *wake duration*), *wake duration* (“In total, how long did these awakenings last?”), and *efficiency* (100% × (duration)/(mins between^“^What time did you get into bed?^”^ and^“^What time did you get out of bed for the day?^”^)).

### 2.4. Analyses

All analyses were conducted blind to treatment group and separately by ITT and PP in IBM SPSS Statistics 28 with the significance threshold set at *p* < .05. Cohen's *d* and *b* estimates are reported as measures of effect size. As these secondary analyses are exploratory, we did not correct for multiple comparisons in pairwise comparisons, instead reporting effect sizes to underscore findings that merit further investigation and replication. Simple Pearson correlations were calculated between change scores (baseline minus end-of-treatment) for overall PTSD symptom severity (PCL total/CAPS total, with and without insomnia/nightmare sleep items, per recommendations (e.g., [30])) and each of the sleep measures (self-reported sleep diary, PTSD sleep symptom severity items, and sleep actigraphy).

While our primary outcomes study was a noninferiority design [25, 26], those analyses are not recommended here since our active control comparison group (CPT) is a first-line treatment for *PTSD* not *sleep* [27]. Instead, we estimated separate fixed linear time, random intercept, mixed models for each of the sleep measures (PTSD nightmares, PTSD insomnia; diary/actigraphy indices for each of quality/fragmentation, latency, duration, wake duration, efficiency). The full models included fixed effects for group (CPT, SKY; coded -0.5, +0.5 for ease of interpretation; [36, 37]), time (baseline, end-of-treatment), and their interaction. Per recommendations, time was mean-centred [36–39], and outliers (≥ ±3 SD) were Winsorized and replaced with the next highest value [40]. (Each of the actigraphy sleep indices had three outliers.) We tested only the simplest covariance structure (compound symmetry; CS) as all repeated measures had only two time points (baseline, end-of-treatment; [39]), and within-subject correlations over time were moderate-to-large and positive [41]. Per recommendations, sleep measure variances at each time point were consistent (i.e., homogeneity of group variances assumption was met), and examination of residual plots showed that models the met assumptions of normality, linearity, independence, and constant variance [41].

## 3. Results

### 3.1. PTSD Sleep Symptoms

#### 3.1.1. Nightmares

**Figure 1:**
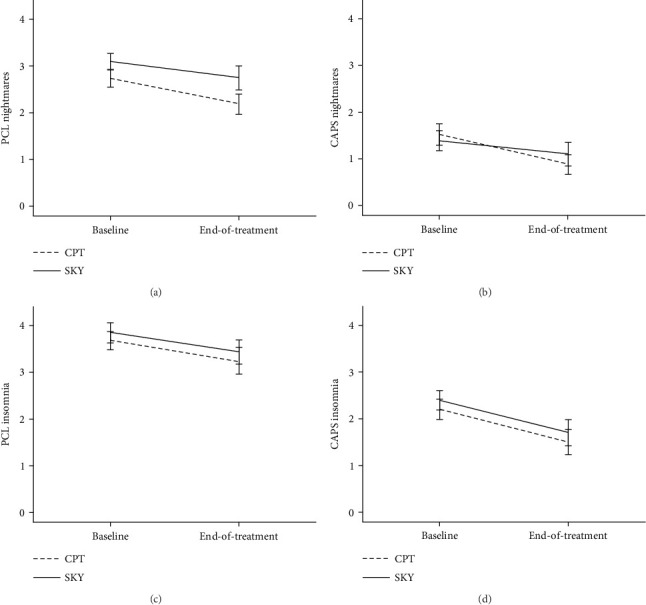
Mean PTSD sleep symptom scores at baseline and end-of-treatment for veterans who received either cognitive processing therapy (CPT) or Sudarshan kriya yoga (SKY) for PTSD (ITT analyses). (a) PCL (self-reported) nightmares. (b) CAPS (clinician-assessed) nightmares. (c) PCL (self-reported) insomnia. (d) CAPS (clinician-assessed) insomnia. Scores of ≥ 3 on the PCL [32] and ≥2 on the CAPS [33] are considered clinically significant.

There was a main effect of time (baseline vs. end-of-treatment) for nightmares on both PCL (ITT: *p* = .006, Cohen's *d* = .34; PP: *p* = .011, Cohen's *d* = .35) and CAPS (ITT: *p* = .035, Cohen's *d* = .18; PP: *p* = .096, Cohen's *d* = .20). While there were no main or interaction effects of group, pairwise comparisons revealed that CPT (PCL: Cohen's *d* = .34; Figure 1(a); CAPS: Cohen's *d* = .34; Figure 1(b); Table 2) tended to demonstrate reductions in nightmares more reliably than SKY, especially on the CAPS (PCL: Cohen's *d* = .32; CAPS: Cohen's *d* = .03).

#### 3.1.2. Insomnia

There was a significant main effect of time (baseline vs. end-of-treatment) for insomnia on both PCL (ITT: *p* = .024, Cohen's *d* = .27; PP: *p* = .036, Cohen's *d* = .28) and CAPS (ITT: *p* = .002, Cohen's *d* = .37; PP: *p* = .009, Cohen's *d* = .35). While there were no main or interaction effects of group, pairwise comparisons revealed that SKY (PCL: Cohen's *d* = .44; Figure 1(c); CAPS: Cohen's *d* = .45; Figure 1(d); Table 2) tended to demonstrate reductions in insomnia more reliably than CPT, especially on the PCL (PCL: Cohen's *d* = .12; CAPS: Cohen's *d* = .31).

### 3.2. Objective Sleep (Actigraphy)

**Figure 2:**
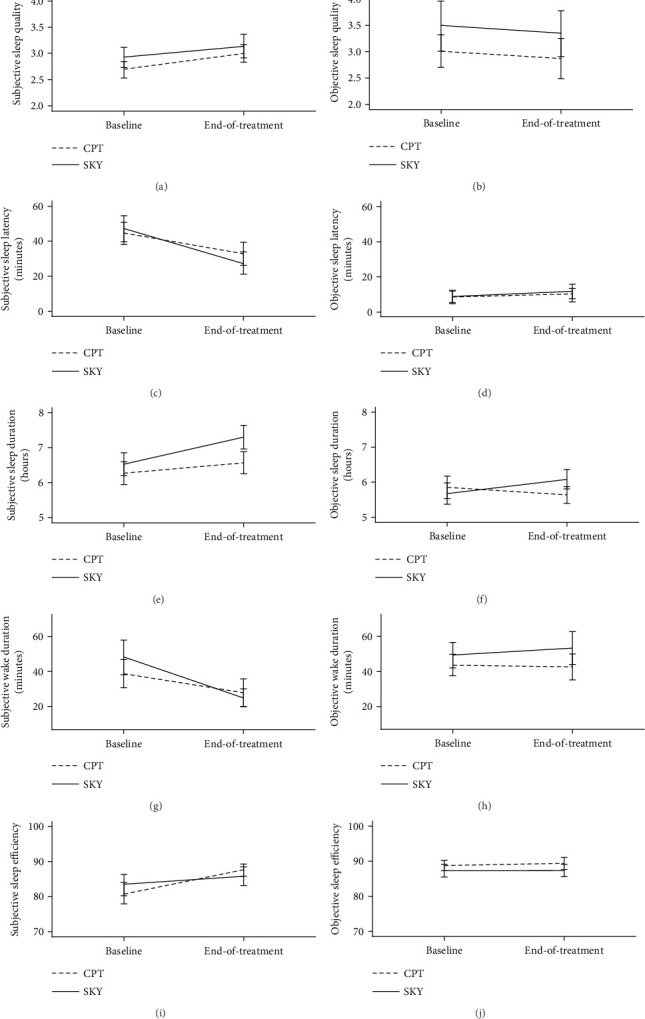
Mean sleep diary and actigraphy scores at baseline and end-of-treatment for veterans who received either cognitive processing therapy (CPT) or Sudarshan kriya yoga (SKY) for PTSD (ITT analyses). (a) Self-reported sleep quality. (b) Actigraphic sleep quality. (c) Self-reported sleep latency (mins). (d) Actigraphic sleep latency (mins). (e) Self-reported sleep duration (hours). (f) Actigraphic sleep duration (hours). (g) self-reported wake duration (mins). (h) actigraphic wake duration (mins). (i) Self-reported sleep efficiency (%). (j) Actigraphy sleep efficiency (%).

There were no significant main or interaction effects of group (CPT vs. SKY) or time (baseline vs. end-of-treatment) for any of the actigraphic sleep indices (Figure 2; Table 3). Exploratory pairwise comparisons revealed inconsistent findings: CPT demonstrated small-to-moderate effect size improvements for sleep efficiency (ITT: Cohen's *d* = −.28; PP: Cohen's *d* = −.28) and wake duration (ITT: Cohen's *d* = .21; PP: Cohen's *d* = .19), while SKY demonstrated a small-to-moderate effect size *worsening* for sleep latency (i.e., longer; ITT: Cohen's *d* = −.33; PP: Cohen's *d* = −.35). For sleep fragmentation (objective “quality”), CPT demonstrated small-to-moderate effect size improvements (ITT: Cohen's *d* = .43; PP: Cohen's *d* = .37), while SKY demonstrated small-to-moderate effect size *worsening* (ITT: Cohen's *d* = −.23; PP: Cohen's *d* = −.25).

### 3.3. Subjective Sleep (Diary)

Overall, both CPT and SKY interventions were associated with improvements in self-reported sleep, with no differences between groups. Findings were similar across both ITT and PP analyses.

#### 3.3.1. Quality

There was a nonsignificant trend in the main effect of time (baseline vs. end-of-treatment) for subjective sleep quality in the ITT sample (*p* = .057; Cohen's *d* = −.25) that reached significance among the PP sample (*p* = .029; Cohen's *d* = .26). Pairwise comparisons confirmed that both CPT (ITT: Cohen's *d* = −.24; PP: Cohen's *d* = −.24; Figure 2(a); Table 4) and SKY (ITT: Cohen's *d* = −.26; PP: Cohen's *d* = −.28; Figure 2(a); Table 4) demonstrated small effect size treatment-related improvements in self-reported sleep quality, and this improvement did not differ by the treatment group.

#### 3.3.2. Latency

There was a significant main effect of time (baseline vs. end-of-treatment) for subjective sleep latency (ITT: *p* = .008, Cohen's *d* = .31; PP: *p* = .008, Cohen's *d* = .31). Pairwise comparisons confirmed that both CPT (ITT: Cohen's *d* = .28; PP: Cohen's *d* = .35; Figure 2(c); Table 4) and SKY (ITT: Cohen's *d* = .30; PP: Cohen's *d* = .27; Figure 2(c); Table 4) demonstrated small effect size treatment-related reductions in self-reported sleep latency, and this improvement did not differ by the treatment group.

#### 3.3.3. Duration

There was a trend main effect of time (baseline vs. end-of-treatment) for subjective sleep total duration in the ITT sample (*p* = .054, Cohen's *d* = -.21) but not in the PP sample (*p* = .102, Cohen's *d* = −.16), suggesting both CPT and SKY were associated with trend-level, small effect size, and treatment-related increases in self-reported total sleep time, though this effect was not evident for treatment completers. Although there were no group main or interaction effects, exploratory pairwise comparisons revealed that only the SKY group demonstrated consistent improvements (ITT: Cohen's *d* = −.29; PP: Cohen's *d* = −.28; Figure 2(e); Table 4); CPT effect sizes were null (ITT: Cohen's *d* = −.13; PP: Cohen's *d* = −.07; Figure 2(e); Table 4).

#### 3.3.4. Wake Duration

There was a significant main effect of time (baseline vs. end-of-treatment) for subjective sleep wake duration (ITT: *p* = .017, Cohen's *d* = .30; PP: *p* = .004, Cohen's *d* = .37). Pairwise comparisons confirmed that both CPT (ITT: Cohen's *d* = .17; PP: Cohen's *d* = .37; Figure 2(g); Table 4) and SKY (ITT: Cohen's *d* = .39; PP Cohen's *d* = .37; Figure 2(g); Table 4) demonstrated small effect size treatment-related reductions in self-reported total time awake during the night, and this improvement did not differ by the treatment group.

There were no significant main or interaction effects of group (CPT vs. SKY) or time (baseline vs. end-of-treatment) for subjective/self-reported sleep efficiency (Figure 2(i); Table 4).

### 3.4. Associations between Treatment-Related Change in PTSD Symptom Severity and Sleep

Correlations are presented in Supplementary Tables 1 and 2. Overall, sleep actigraphy failed to show any statistically significant treatment-related associations with overall PTSD symptom severity. However, sleep as measured by the PTSD sleep symptoms and sleep diary showed numerous statistically significant correlations with overall PTSD symptom severity. The directions of the associations were in the expected direction: improvements in sleep following either CPT or SKY were positively associated with treatment-related improvements in global PTSD severity. The strongest associations were between PCL/CAPS sleep symptoms and total PCL/CAPS. For the sleep diary measures, following CPT, associations were strongest between PCL total and sleep quality, latency, and duration change scores, and following SKY, they were strongest between CAPS total and sleep latency, duration, number of awakenings, and wake duration change scores.

## 4. Discussion

To our knowledge, this is the first study to investigate sleep as an outcome of CPT or SKY for PTSD across a combination of subjective diary, PTSD sleep symptom severity, and objective actigraphic measures. Across subjective sleep measures, we found significant treatment-related improvements in self-reported sleep quality, shorter sleep latency, less total time awake during the night, fewer nightmares, and less insomnia, for both CPT and SKY. However, there were no statistically significant effects of either CPT or SKY for any of the objective actigraphy-measured sleep indices.

Improvements in subjective sleep measures following CPT lend support to a growing body of evidence that trauma-focused psychotherapy for PTSD improves sleep alongside PTSD symptoms. Previous studies examined either female-only samples [13, 15], chronic PTSD linked to multiple traumas [16], or actively serving Military/Defence personnel [14]. Our findings confirm that these treatment-related subjective sleep improvements extend to a sample of predominantly male ex-serving Military/Defence (i.e., veterans). Interestingly, SKY, a regulated, cyclical, yoga breathing meditation, also produced improvements in subjective sleep measures. To date, much of the literature on yoga for sleep has focused on cancer [42, 43], older adults [44], or healthy adults [45]. Here, we extend these findings to suggest that yoga-based interventions may also be beneficial for subjective sleep among individuals with emotional or mental health disorders like PTSD. Given the high drop-out rates associated with trauma-focused psychotherapy, these positive sleep effects for SKY further enhance its promise as a PTSD treatment. As sleep improvements are associated with resilience [46] and general physical and mental wellness [5, 47–50], future research might investigate whether psychological and mind-body interventions like CPT and SKY could be delivered to both active and returned military/defence personnel during recruitment training and discharge to build resilience and protect against future psychopathology and/or physical health problems.

Despite statistically significant improvements in subjective diary and PTSD symptom severity sleep measures, we did not achieve full remission of all sleep disturbances following either CPT or SKY. First, in our study, PTSD insomnia symptoms remained at end-of-treatment, on average, within a range considered clinically significant as measured by the PCL (≥3; [32]) but not by the CAPS (≥2; [33]). Second, total sleep duration was, on average, below recommendations for adults (<7-9 hrs; [35, 51]). However, on average, at end-of-treatment across both groups, PTSD nightmares were *below* clinical threshold on both PCL and CAPS, subjective sleep quality was fair, and sleep latency and efficiency were at clinical cut-off for insomnia (30 min and 85%, respectively; [52]), whereas at baseline, they were closer to (though slightly better than) ratings seen in individuals with insomnia disorder (49 min and 70%, respectively; [35]). Overall, this suggests fair-to-good sleep on these indices (though it is worth noting that PTSD nightmares were, on average, at or just below the threshold for both groups at baseline). These general sleep improvements are consistent with the PTSD, depression, and negative affect improvements we observed with both SKY and CPT in the primary outcomes study [25]. The presence of residual sleep disturbance symptoms despite improvements in PTSD severity is consistent with extant literature [13–15] and aligns with our primary outcomes study where many veterans retained significant residual PTSD symptoms despite overall treatment-related improvements (an effect commonly reported in PTSD treatment research; [53, 54]). In particular, PTSD insomnia symptoms commonly remain despite improvements on PTSD nightmares and self-reported sleep diary measures [16], which has led some researchers to speculate this residual insomnia represents a distinct disorder rather than secondary PTSD symptoms [14]. As poor sleep is a risk factor for PTSD relapse [6–8] and poor mental and physical health more generally [5, 47–50], it is critical for PTSD interventions to target sleep. A recent meta-analysis of RCTs suggests that cognitive behavioural therapy for insomnia (CBT-I) improves PTSD symptoms at similar effect sizes to first-line PTSD treatments [55], yet all comparisons were to wait-list controls. Future research should investigate head-to-head comparisons of CBT-I to trauma-focused psychotherapies and assess for noninferiority for both sleep and PTSD outcomes, as well as for any potential additive or interactive effects of their combinations. These studies should also explore the potential moderating effects of comorbid insomnia disorder and comorbid sleep apnoea.

We did not observe statistically significant changes in any of the objective sleep indices following CPT or SKY. While exploratory pairwise comparisons revealed some (nonsignificant) small-to-moderate effect size within-group changes, these effects were not apparent in data visualisations, and inconsistent findings concealed any clear pattern in the data. Overall, this suggests a high level of variability in the objective sleep indices rather than any true group or treatment-related differences, possibly due to the high proportion of likely sleep apnoea in our sample [56] which may obscure any treatment-related effects. Alternatively, our lack of significant treatment-related objective sleep effects might be due to floor effects: the objective sleep indices in our study, both at baseline and end-of-treatment, were consistent with those of objectively assessed “good sleepers” (sleep duration >5.37 hrs; [35]) and/or better than the objective clinical cut-offs for insomnia (sleep latency <20 min, sleep efficiency >80%; [57]), suggesting no clinically-significant actigraphy-measured sleep difficulties in our sample. Indeed, a diagnosis of insomnia disorder requires only a (self-reported) *dissatisfaction* with sleep quality/quantity [1], not formal confirmation via objective measures, and our findings are consistent with several studies that have failed to find agreement between subjective and objective sleep measures [35, 58, 59]. Specifically, one noninferiority RCT that compared CBT-I to Tai Chi Chih (i.e., a treatment design similar to our study, comparing a psychological to a complementary and integrative health intervention) found improvements in self-reported but not polysomnographic sleep across both groups [60]. A failure to find treatment-related improvements in objective measures adds further weight to the argument that it may be more the *perceived* (i.e., subjective) disruptions to sleep that underpin clinical mental health disorders, rather than objective disruptions. A growing body of evidence demonstrates that maladaptive beliefs about sleep may drive the development and maintenance of insomnia and influence self-report ratings of sleep [61, 62], especially in individuals with emotional disorders like PTSD [63–65]. CBT-I, the gold standard evidence-based intervention for sleep, targets both poor sleep hygiene and maladaptive beliefs about sleep, and at least one study has used actigraphy to inform behavioural experiments and shift sleep cognitions in the context of therapy for individuals with insomnia [66]. While our RCT employed a trauma-focused psychotherapy (CPT) that does not specifically target sleep cognitions, it is possible that by targeting beliefs about the trauma, veterans were able to transfer these reappraisal skills to other domains such as sleep beliefs, thus leading to improvement in self-reported sleep. Yoga practices like SKY typically cultivate mindfulness which could lead to similar enhancement of reappraisal skills, possibly indirectly via increased acceptance [67]. While the supplementary correlation analyses are consistent with these inferences, mechanistic studies that explore reappraisal and acceptance as mediators of treatment outcome are needed to confirm these hypotheses.

The main strength of our study is the use of both subjective and objective sleep measures to gain a more comprehensive understanding of sleep responses to CPT or SKY for PTSD. Furthermore, we used both standardised sleep-specific diary measures and more general PTSD insomnia/nightmare sleep symptom items to increase the reliability of subjective self-report measures and to allow direct comparison with previous studies that have used only one of these self-report measures. A clear picture emerged of consistent improvements across subjective diary and PTSD symptom severity sleep measures for both CPT and SKY. Yet, inconsistencies between subjective and objective measures and subtle differences in whether improvements reached clinical threshold support continued use of multiple measures in clinical treatment studies involving PTSD.

A limitation of our study is that the sleep diary and actigraphy measures were taken from only a single night's sleep at each baseline and end-of-treatment. Assessing 1-2 continuous weeks of data tends to increase both their independent reliabilities [61, 68] and the likelihood of strong concordance between the two [35], especially for latency [57]. Moreover, night-to-night variability in sleep is more strongly related to subjective than objective measures [69]. We chose a single night's sleep to reduce participant burden [70], given that the high number of treatment-related assessments in our RCT. The consistency of findings across measures with varying timeframes (sleep diary rated across one night, CAPS and PCL rated across the previous month) increases confidence in their reliability. Furthermore, all overnight sleep assessments were taken on weekday nights; therefore, we reduced the likelihood of impact from weekday versus weeknight variabilities [71, 72]. However, it is possible that the single-night assessment period accounts for our inconsistent and nonsignificant actigraphy effects. Similarly, another limitation is that we did not conduct a comprehensive sleep evaluation (e.g., presence of diagnosed mental or physical sleep disorders, use of sleep medications). Self-report measures suggest, on average, the veterans in both the CPT and SKY groups likely had sleep apnoea, with no differences between groups. This high prevalence rate is common among individuals with PTSD, especially veterans [73]. However, it may also explain the lack of concordance between our self-report and actigraphy sleep measures, as there is some suggestion that actigraphy precision may be lower for single night assessment of individuals with sleep apnoea [56]. Future research should replicate our study using a longer sleep assessment period (e.g., 1-2 weeks); a more general sleep measure like the Insomnia Severity Index (past 2 weeks; [74]), Pittsburgh Sleep Quality Index (past month; [75]), or Sleep Timing Questionnaire (normal average week; [70]); and conduct sensitivity analyses based on presence/absence of sleep disorders (using clinician administered or objective tests) and use of sleep medications. We also acknowledge our use of the outdated PCL-C due to RCT design parameters [25, 26] and recommend future studies replicate using the PCL-5, though there is no difference between the two measures (nor DSM criteria) for the insomnia/nightmare items.

## 5. Conclusion

To our knowledge, this is the first study to investigate sleep as an outcome of either CPT or SKY for PTSD across a combination of subjective diary, PTSD symptom severity, and objective actigraphic measures. We found significant treatment-related improvements in self-reported and clinician-administered measures of sleep quality, shorter sleep latency, less total time awake during night, fewer nightmares, and less insomnia for both CPT and SKY. In contrast, we did not observe significant changes in any of the objective (actigraphy) sleep indices for either CPT or SKY. The similarities in sleep-related improvements between CPT and SKY lend support to a growing body of evidence that trauma-focused psychotherapy for PTSD improves sleep and suggest that yoga-based interventions may also be beneficial for sleep among individuals with emotional or mental health disorders like PTSD.

## Figures and Tables

**Table 1:** Baseline demographics and clinical characteristics by treatment group across samples.

	ITT		PP		Valid Actigraphy	
	CPT (*n* = 44)	SKY (*n* = 41)	CPT (*n* = 29)	SKY (*n* = 30)	CPT (*n* = 37)	SKY (*n* = 36)
Age	56.4 (12.9)	57.4 (12.6)	58.2 (13.0)	60.7 (11.0)	56.2 (13.5)	58.3 (11.8)
% male/female	93.2/6.8	82.9/17.1	93.1/6.9	76.8/23.2	91.9/8.1	83.3/16.7
% white	65.9	53.7	65.5	60.0	67.6	52.8
% married or domestic partner	45.4	34.1	41.4	36.7	43.2	36.1
% bachelor's degree or higher	31.8	24.4	27.5	23.3	35.1	22.2
Total CAPS-5	34.1 (14.4)	32.3 (14.2)	31.6 (14.2)	30.1 (12.5)	34.1 (14.9)	33.2 (14.7)
Adjusted CAPS-5^a^	30.4 (12.7)	28.5 (12.9)	28.5 (12.7)	26.5 (11.2)	30.2 (13.2)	29.4 (13.4)
Total PCL-C	56.2 (11.7)	56.9 (13.6)	49.6 (9.0)	53.6 (11.6)	55.7 (11.6)	56.4 (14.2)
Adjusted PCL-C^a^	49.8 (10.2)	50.0 (12.3)	47.7 (10.4)	49.0 (11.8)	49.2 (10.2)	49.4 (12.9)
Total PCL-5	52.7 (10.4)	55.4 (11.7)	49.6 (9.0)	53.6 (11.6)	51.6 (9.9)	55.8 (11.7)
Adjusted PCL-5^a^	47.7 (9.5)	49.9 (10.8)	45.2 (8.3)	48.1 (10.9)	46.5 (9.2)	50.0 (11.1)
% comorbid MDD^b^	43.2	48.8	41.4	43.3	37.8	52.8
% comorbid anxiety disorder^b^	61.4	58.5	55.2	60.0	56.8	58.3
% comorbid AUD^b^	20.5	19.5	13.8	13.3	18.9	22.2
Probability of sleep apnoea^c^	.69 (.22)	.66 (.23)	.69 (.23)	.66 (.24)	.69 (.23)	.66 (.22)
Restless Legs Score^c^	-12.4 (15.6)	-9.0 (18.4)	-11.8 (17.4)	-10.0 (18.3)	-13.1 (15.5)	-7.6 (18.2)

Note. ITT = intent-to-treat; PP = per protocol; CPT = cognitive processing therapy; SKY = Sudarshan kriya yoga; CAPS-5 = Clinician-Administered PTSD Scale for DSM-5; PCL-C = PTSD Checklist–Civilian Version (outcome measure); PCL-5 = PTSD Checklist for DSM-5 (screening measure). Except where indicated by %, values are presented in the format M (SD), where M = mean and SD = standard deviation. ^a^Insomnia and nightmare items removed, per recommendations (e.g., [30]). ^b^Assessed via the Mini International Neuropsychiatric Interview for DSM-5 (MINI; [31]). MDD = major depressive disorder; AUD = alcohol use disorder. Anxiety disorder includes any of generalised anxiety, social anxiety, panic, or agoraphobia disorders. ^c^Assessed via the MAPI and RLS-DI, respectively.

**Table 2:** PTSD sleep symptom effects for time and group by time for both ITT and PP analyses.

PTSD sleep symptom index	Time						Group × time					
	ITT (base *n* = 85; EOT *n* = 64)			PP (*N* = 59)			ITT (base *n* = 85; EOT *n* = 64)			PP (*N* = 59)		
	*b*	*se*	*t*	*b*	*se*	*t*	*b*	*se*	*t*	*b*	*se*	*t*
PCL insomnia	-.04⁣^∗^	.02	-2.31	-.04⁣^∗^	.02	0.04	-.03	.04	-0.82	-.05	.04	-1.30
CAPS insomnia	-.08⁣^∗∗^	.03	-3.28	-.07⁣^∗∗^	.03	-2.72	-.01	.05	-0.12	-.01	.05	-0.15
PCL nightmares	-.05⁣^∗^	.02	-2.87	-.04⁣^∗^	.02	-2.63	.01	.03	0.18	-.01	.03	-0.33
CAPS nightmares	-.04⁣^∗^	.02	-2.16	-.03^	.02	-1.69	.04	.04	1.19	.02	.04	0.521

Note. PTSD = posttraumatic stress disorder; ITT = intent-to-treat; base = baseline; EOT = end-of-treatment; PP = per protocol. ⁣^∗∗^*p* < .01; ⁣^∗^*p* < .05; ^*p* = .05 − .10.

**Table 3:** Objective sleep (actigraphy) effects for time and group by time for both ITT and PP analyses.

Actigraphy sleep index	Time						Group × time					
	ITT (base *n* = 73; EOT *n* = 58)			PP (base *n* = 51; EOT *n* = 53)			ITT (base *n* = 73; EOT *n* = 58)			PP (base *n* = 51; EOT *n* = 53)		
	*b*	se	*t*	*b*	se	*t*	*b*	se	*t*	*b*	se	*t*
Fragmentation	-0.02	0.03	-0.68	-0.02	0.04	-0.49	0.09	0.06	1.44	0.10	0.07	1.32
Latency	0.28	0.45	0.62	0.51	0.45	1.14	0.34	0.90	0.38	0.29	0.90	0.32
Duration	0.55	1.88	0.29	0.96	2.08	0.46	3.71	3.76	0.99	0.84	4.16	0.20
Number awaken	-0.04	0.10	-0.35	0.01	0.11	0.08	0.23	0.20	1.13	0.20	0.22	0.91
Wake duration	0.11	0.72	0.16	0.26	0.79	0.33	1.11	1.43	0.77	1.66	1.57	1.06
Efficiency	0.05	0.17	0.26	0.02	0.19	0.12	-0.22	0.35	-0.64	-0.39	0.39	-1.01

*Note*. ITT = intent-to-treat; base = baseline; EOT = end-of-treatment; PP = per protocol.

**Table 4:** Subjective sleep (diary) effects for time and group by time for both ITT and PP analyses.

Sleep diary index	Time						Group × time					
	ITT (base *n* = 79; EOT *n* = 60)			PP (*N* = 55)			ITT (base *n* = 79; EOT *n* = 60)			PP (*N* = 55)		
	*b*	se	*t*	*b*	se	*t*	*b*	se	*t*	*b*	se	*t*
Quality	0.04^	0.02	1.91	0.04⁣^∗^	0.02	2.19	-0.00	0.04	-0.08	0.02	0.04	0.49
Latency	-1.73⁣^∗∗^	0.64	-2.72	-2.01⁣^∗∗^	0.74	-2.74	-0.65	1.27	-0.52	-0.47	1.47	-0.32
Duration	0.06^	0.03	1.93	0.07	0.04	1.63	0.05	0.07	0.80	0.08	0.08	0.95
Number awaken	-0.07^	0.04	-1.86	-0.07	0.04	-1.65	-0.05	0.08	-0.69	-0.02	0.09	-0.25
Wake duration	-2.09⁣^∗^	0.87	-2.41	-2.75	0.96	-2.87⁣^∗∗^	-1.57	1.73	-0.91	-1.35	1.92	-0.70
Efficiency	0.13	0.35	0.38	-0.28	0.31	-0.91	0.22	0.69	0.31	1.11^	0.62	1.77

*Note*. ITT = intent-to-treat; base = baseline; EOT = end-of- treatment; PP = per protocol. ⁣^∗∗^*p* < .01; ⁣^∗^*p* < .05; ^*p* = .05 − .10.

## Data Availability

The datasets generated and/or analysed during the current study are not publicly available due to institutional regulations protecting service member data but are available from the corresponding author on reasonable written request (may require data use agreements to be developed).

## Supplementary Materials

Supplementary 1 CONSORT 2010 checklist of information for the RCT.

Supplementary 2 Supplementary tables: simple Pearson correlations between each of the sleep measures (self-reported sleep diary, PTSD sleep symptom severity items, and sleep actigraphy) and overall PTSD symptom severity change scores (baseline minus end-of-treatment; PCL total/CAPS total, with and without insomnia/nightmare sleep items, per recommendations).
