# Supplementary material for: US Veterans Show Improvements in Subjective but Not Objective Sleep following Treatment for Posttraumatic Stress Disorder: Secondary Analyses from a Randomised Controlled Trial
Source: Depress Anxiety. 2023 Aug 14;2023:7001667. doi: 10.1155/2023/7001667 (PMC11922038; doi:10.1155/2023/7001667)
Supplement: Supplementary 2 — Supplementary tables: simple Pearson correlations between each of the sleep measures (self-reported sleep diary, PTSD sleep symptom severity items, and sleep actigraphy) and overall PTSD symptom severity change scores (baseline minus end-of-treatment; PCL total/CAPS total, with and without insomnia/nightmare sleep items, per recommendations). [file 7001667.f2.docx]

Supplementary Table 1

*Pearson correlations between sleep and PTSD change scores (baseline minus end-of-treatment)*

| Sleep Measure | CPT | | | |  | SKY | | | |
| --- | --- | --- | --- | --- | --- | --- | --- | --- | --- |
|  | *ITT* | | *PP* | |  | *ITT* | | *PP* | |
|  | PCL Total | CAPS Total | PCL Total | CAPS Total |  | PCL Total | CAPS Total | PCL Total | CAPS Total |
| PTSD Sleep Symptom Index |  |  |  |  |  |  |  |  |  |
| PCL Insomnia | .49** | 0.34^ | .50** | 0.36^ |  | .42* | 0.26 | .41* | 0.27 |
| CAPS Insomnia | 0.22 | .65*** | 0.26 | .67*** |  | 0.12 | .57*** | 0.25 | .56** |
| PCL Nightmares | .51** | .40* | .49** | .40* |  | .58*** | 0.32^ | .59*** | 0.33^ |
| CAPS Nightmares | -0.06 | 0.14 | -0.11 | 0.16 |  | 0.33^ | .38* | 0.34^ | .39* |
| Actigraphy Sleep Index |  |  |  |  |  |  |  |  |  |
| Fragmentation | -0.15 | -0.18 | -0.18 | -0.20 |  | -0.12 | 0.09 | -0.12 | 0.11 |
| Latency | -0.07 | 0.13 | -0.09 | 0.17 |  | 0.14 | -0.11 | 0.09 | -0.06 |
| Duration | 0.33 | 0.18 | 0.23 | 0.20 |  | 0.06 | -0.07 | 0.12 | -0.09 |
| Number Awaken | -0.03 | -0.08 | -0.11 | -0.12 |  | -0.11 | 0.08 | -0.04 | 0.08 |
| Wake Duration | 0.00 | -0.15 | -0.03 | -0.15 |  | -0.06 | 0.11 | 0.03 | 0.08 |
| Efficiency | 0.05 | 0.25 | 0.07 | 0.24 |  | 0.14 | -0.11 | 0.08 | -0.09 |
| Sleep Diary Index |  |  |  |  |  |  |  |  |  |
| Quality | -.40* | -0.13 | -.41* | -0.10 |  | -0.05 | -0.13 | -0.07 | -0.10 |
| Latency | 0.24 | 0.35^ | 0.34^ | .40* |  | 0.08 | 0.29 | 0.13 | 0.27 |
| Duration | -.36* | -0.25 | -0.31 | -0.23 |  | 0.14 | 0.25 | 0.13 | 0.27 |
| Number Awaken | 0.09 | -0.10 | 0.10 | -0.08 |  | 0.10 | 0.34 | 0.04 | .42* |
| Wake Duration | -0.19 | -0.04 | -0.05 | -0.02 |  | -0.14 | -0.24 | -0.16 | -0.23 |
| Efficiency | 0.11 | -0.03 | 0.11 | -0.05 |  | 0.08 | -0.01 | 0.08 | -0.02 |

*Note*. ITT = intent-to-treat; PP = per protocol; CPT = cognitive processing therapy; SKY = Sudarshan kriya yoga. *** *p* < .001, ** *p* < .01, * *p* < .05, ^ *p* = .05-.10.

Supplementary Table 2

*Pearson correlations between sleep and adjusted PTSD change scores (baseline minus end-of-treatment)*

| Sleep Measure | CPT | | | |  | SKY | | | |
| --- | --- | --- | --- | --- | --- | --- | --- | --- | --- |
|  | *ITT* | | *PP* | |  | *ITT* | | *PP* | |
|  | Adjusted PCL Total^a^ | Adjusted CAPS Total^a^ | Adjusted PCL Total^a^ | Adjusted CAPS Total^a^ |  | Adjusted PCL Total^a^ | Adjusted CAPS Total^a^ | Adjusted PCL Total^a^ | Adjusted CAPS Total^a^ |
| PTSD Sleep Symptom Index |  |  |  |  |  |  |  |  |  |
| PCL Insomnia | .395* | 0.347^ | .400* | 0.366^ |  | 0.328^ | 0.206 | 0.32^ | 0.217 |
| CAPS Insomnia | 0.222 | .581*** | 0.262 | .603*** |  | 0.088 | .452* | 0.224 | .449* |
| PCL Nightmares | .439* | .399* | .416* | .385* |  | .508*** | 0.293 | .518*** | 0.305 |
| CAPS Nightmares | -0.113 | 0.085 | -0.168 | 0.112 |  | 0.28 | 0.259 | 0.293 | 0.265 |
| Actigraphy Sleep Index |  |  |  |  |  |  |  |  |  |
| Fragmentation | -0.139 | -0.16 | -0.174 | -0.182 |  | -0.111 | 0.074 | -0.115 | 0.084 |
| Latency | -0.099 | 0.164 | -0.117 | 0.206 |  | 0.164 | -0.116 | 0.097 | -0.085 |
| Duration | 0.344^ | 0.156 | 0.232 | 0.175 |  | 0.081 | -0.036 | 0.145 | -0.049 |
| Number Awaken | -0.007 | -0.07 | -0.091 | -0.11 |  | -0.087 | 0.071 | -0.025 | 0.069 |
| Wake Duration | 0.027 | -0.131 | -0.006 | -0.127 |  | -0.098 | 0.077 | -0.011 | 0.056 |
| Efficiency | 0.03 | 0.219 | 0.049 | 0.206 |  | 0.174 | -0.073 | 0.117 | -0.056 |
| Sleep Diary Index |  |  |  |  |  |  |  |  |  |
| Quality | -.405* | -0.138 | -.424* | -0.102 |  | -0.05 | -0.117 | -0.079 | -0.099 |
| Latency | 0.231 | 0.326^ | 0.333^ | .374* |  | 0.102 | 0.323 | 0.161 | 0.309 |
| Duration | -0.341^ | -0.247 | -0.286 | -0.221 |  | 0.151 | 0.292 | 0.139 | 0.307 |
| Number Awaken | 0.118 | -0.106 | 0.133 | -0.082 |  | 0.106 | 0.348^ | 0.034 | 0.404 |
| Wake Duration | -0.202 | -0.059 | -0.056 | -0.059 |  | -0.138 | -0.202 | -0.157 | -0.196 |
| Efficiency | 0.128 | -0.008 | 0.131 | -0.024 |  | 0.115 | -0.016 | 0.122 | -0.02 |

*Note*. ITT = intent-to-treat; PP = per protocol; CPT = cognitive processing therapy; SKY = Sudarshan kriya yoga. *** *p* < .001, ** *p* < .01, * *p* < .05, ^ *p* = .05-.10.

^a^ Insomnia and nightmare items removed, per recommendations ([e.g., Pigeon et al., 2013](#_ENREF_1)).
